# Non-thermal plasma modulates cellular markers associated with immunogenicity in a model of latent HIV-1 infection

**DOI:** 10.1371/journal.pone.0247125

**Published:** 2021-03-01

**Authors:** Hager Mohamed, Ramona Clemen, Eric Freund, Jan-Wilm Lackmann, Kristian Wende, Jennifer Connors, Elias K. Haddad, Will Dampier, Brian Wigdahl, Vandana Miller, Sander Bekeschus, Fred C. Krebs

**Affiliations:** 1 Department of Microbiology and Immunology, Institute for Molecular Medicine & Infectious Disease, Drexel University College of Medicine, Philadelphia, Pennsylvania, United States of America; 2 Centre for Innovation Competence (ZIK) *plasmatis*, Leibniz Institute for Plasma Science and Technology Greifswald (INP), Greifswald, Germany; 3 CECAD proteomics facility, University of Cologne, Cologne, Germany; 4 Division of Infectious Diseases and HIV Medicine, Drexel University College of Medicine, Philadelphia, Pennsylvania, United States of America; George Mason University, UNITED STATES

## Abstract

Effective control of infection by human immunodeficiency virus type 1 (HIV-1), the causative agent of the acquired immunodeficiency syndrome (AIDS), requires continuous and life-long use of anti-retroviral therapy (ART) by people living with HIV-1 (PLWH). In the absence of ART, HIV-1 reemergence from latently infected cells is ineffectively suppressed due to suboptimal innate and cytotoxic T lymphocyte responses. However, ART-free control of HIV-1 infection may be possible if the inherent immunological deficiencies can be reversed or restored. Herein we present a novel approach for modulating the immune response to HIV-1 that involves the use of non-thermal plasma (NTP), which is an ionized gas containing various reactive oxygen and nitrogen species (RONS). J-Lat cells were used as a model of latent HIV-1 infection to assess the effects of NTP application on viral latency and the expression of pro-phagocytic and pro-chemotactic damage-associated molecular patterns (DAMPs). Exposure of J-Lat cells to NTP resulted in stimulation of HIV-1 gene expression, indicating a role in latency reversal, a necessary first step in inducing adaptive immune responses to viral antigens. This was accompanied by the release of pro-inflammatory cytokines and chemokines including interleukin-1β (IL-1β) and interferon-γ (IFN-γ); the display of pro-phagocytic markers calreticulin (CRT), heat shock proteins (HSP) 70 and 90; and a correlated increase in macrophage phagocytosis of NTP-exposed J-Lat cells. In addition, modulation of surface molecules that promote or inhibit antigen presentation was also observed, along with an altered array of displayed peptides on MHC I, further suggesting methods by which NTP may modify recognition and targeting of cells in latent HIV-1 infection. These studies represent early progress toward an effective NTP-based *ex vivo* immunotherapy to resolve the dysfunctions of the immune system that enable HIV-1 persistence in PLWH.

## Introduction

Human immunodeficiency virus type 1 (HIV-1) is a lentivirus that causes lifelong infection and affects over 30 million people worldwide despite the administration of anti-retroviral therapy (ART). In the absence of ART, patients progress to acquired immune deficiency syndrome (AIDS), which is characterized by a rise in viremia and compromised function of CD4+ T cells, which are the primary targets of HIV-1 replication. Even in patients who have been well suppressed on ART for years, progression to AIDS occurs if ART is discontinued. The inability of patients to control HIV-1 infection in the absence of ART is largely due to the establishment of latent reservoirs in which integrated viral DNA can remain stable in resting CD4+ T cells for years, with greatly decreased display of virus-derived peptides on the infected cell [1–3]. These chronically HIV-1 infected subjects show impairments in both the innate and adaptive immune systems. These include numerous defects in antigen-presenting cells (APCs) that contribute to the decreased engagement with the cells of the adaptive immune response, manifesting as impaired maturation, dysregulated pro-inflammatory cytokine release, and inefficient activation of T lymphocytes [4–8]. Furthermore, targeting of actively infected cells by cytotoxic T lymphocytes (CTLs) is avoided via viral evasion mechanisms such as downregulation of MHC I and costimulatory CD28 on the infected cell [9–11]. Therefore, any therapeutic intervention(s) designed to provide drug-free therapy for HIV-1 infection must modify multiple cellular processes to promote effective control over HIV-1.

In this study, we have taken the first steps toward developing a novel immunomodulatory therapy for HIV-1-infected patients that may simultaneously address several of the immune deficiencies associated with HIV-1 persistence and clinical latency. Our approach utilizes non-thermal plasma (NTP), an ionized gas predominantly composed of various reactive oxygen and nitrogen species (RONS), including hydroxyl ions, superoxide, nitric oxide, and hydrogen peroxide [12,13].

The immunomodulatory effects of NTP have been demonstrated in studies involving different cancers, which share some of the immunological deficiencies characteristic of HIV-1 infection in PLWH. When applied to cells *in vitro*, as can be done using a wide variety of plasma-generating devices, NTP has been shown to stimulate the display or release of multiple damage-associated molecular patterns (DAMPs) that promote APC responses, including chemotaxis and phagocytosis of infected or cancerous cells. The DAMPs reported to be induced in response to NTP include the pro-chemotactic interleukin-1β (IL-1β), high-mobility group box 1 protein (HMGB1), IL-8, the pro-phagocytic calreticulin (CRT), heat shock protein 70 (HSP70), and HSP90 [14–19]. Correlations of many of these markers with effective immune responses were demonstrated in mice challenged with tumor cells that were exposed *in vitro* to NTP; both the innate and adaptive immune responses were stimulated as demonstrated by increased infiltration of different immune cell subsets into tumors, cancer cell-specific systemic CD8+ T cell responses, decreased tumor size and burden, and increased survival of mice with metastatic cancer [15–20].

In addition to its immunogenic effect, NTP is also hypothesized to induce increased antigenicity of virally infected or tumor cells. Application of NTP to melanoma cells was recently shown to increase surface MHC I expression, thus reversing the decreased MHC I expression in cancer cells that facilitates evasion of cytotoxic T lymphocytes (CTL) activity [21]. The ability of NTP to promote the exposure of neoantigens (that would allow for a more effective *de novo* CTL response) capable of inducing effective CTL targeting has also been proposed [22]. To this point, NTP-augmented immunomodulation has not been examined in cellular or animal models of viral diseases, although NTP suppression of infection by different viruses *in vitro*, including HIV-1, has been reported. Due to the potential of NTP to modulate both innate and adaptive immune responses (as reported in *in vivo* studies of various cancers), we investigated NTP in an *in vitro* model of HIV-1 latent infection as an agent capable of overcoming some of the immunodeficiencies that contribute to disease chronicity by increasing the appearance of markers associated with enhanced immunogenicity.

## Materials and methods

### Cell culture

Jurkat cells (clone E6; ATCC: TIB152), J-Lat cells (clone 10.6; NIH AIDS Reagent Program), and THP-1 cells (ATCC: TIB202) were cultured in RPMI supplemented with 10% fetal bovine serum (FBS) and 1% penicillin/streptomycin at 37°C and 5% CO_2_. Cells were maintained in a T75 flask and passaged every 2–4 days.

### Non-thermal plasma application

The atmospheric pressure plasma jet kINPen (neoplas tools) was used as the plasma source. Argon (99.999%, AirLiquide) was used as carrier gas. The plasma was ignited using a high-frequency voltage pin-type electrode within an internal ceramic capillary. The length of the plasma effluent was about 11 mm. J-Lat cells were seeded at 4x10^5^/mL into 24-well plates in 0.5 mL medium per well and directly exposed to NTP at room temperature. Samples in each well were exposed to NTP for different durations for production of different amounts of RONS [12,13]. Following NTP exposure, cells were incubated at 37°C and 5% CO_2_ for at least 24 h.

### Cell surface molecule detection

NTP-exposed cells were incubated for at least 24 h and then assayed for cell surface-associated pro-phagocytic DAMPs, major histocompatibility complex I (MHC I), costimulatory molecules, and immune checkpoint molecules. Cells were washed twice with PBS and then stained with fluorochrome-conjugated antibodies specific to CRT-PE (clone FMC75, Enzo), HSP70-AF488 (clone EPR16892, Abcam), HSP90-AF700 (clone AC88, Novus), HLA-ABC-PE-Cy7 (clone G46-2.6, BD), CD28-APC (clone CD28.2, antibodies-online), CD134-APC-Cy7 (clone Ber-ACT35, BioLegend), CD152-PE (clone BNI3, BioLegend), CD154-APC-Cy7 (clone TRAP1, BD), CD278-BV421 (clone C398.4A, BioLegend), and CD279-AF647 (clone EH12.2H7, BioLegend) at 4°C for 20 min. After incubation with antibodies, cells were washed twice with phosphate buffered saline (PBS) and then stained with 4′,6-diamidino-2-phenylindole (DAPI) at 250 nM for gating of viable cells. Cell surface molecules were detected using a flow cytometer (Gallios, Beckman-Coulter), and data were analyzed using Kaluza 2.1 software (Beckman-Coulter).

### HMGB1 western blot analysis

J-Lat cells were exposed to NTP, and cell-free supernatants were collected by centrifugation 24 h post-incubation. Supernatants were concentrated 6-fold in 10 kDA Amicon tubes (MilliporeSigma) and protein concentrations were measured by the Bradford protein assay using the Pierce protein assay kit (ThermoFisher Scientific, 23225) following manufacturer’s protocol. Released HMGB1 protein was detected using a recombinant rabbit monoclonal antibody (ThermoFisher Scientific, MA5-31967) diluted in TBS-Tween-20 with 1% BSA and anti-rabbit HRP secondary antibody diluted in TBS-Tween-20 supplemented with 10 g milk powder. Total protein was quantified using chemiluminescence detection with the SuperSignal West Dura procedure as previously described by the manufacturer (ThermoFisher Scientific), and protein bands were quantified using ImageJ.

### Mutliplex assay

Jurkat or J-Lat cell supernatants were collected by centrifugation 24 h post-NTP exposure. In separate experiments, Jurkat cells exposed for 15 s to NTP were incubated for 24 h and then co-cultured with THP-1 cells for 1 h before supernatants were collected. Supernatants were assayed for IL-1β, IL-4, IL-6, IL-8, IL-10, IL-13, IL-18, chemokine ligand 2 (CCL2), tumor necrosis factor-α (TNF-α), and IFN-γ using multiplex bead technology (BioLegend). Data were acquired using flow cytometry (CytoFLEX S, Beckman-Coulter) and analyzed using the appropriate analysis software (VigeneTech). Absolute concentrations (pg/mL) were calculated from an asymmetric sigmoidal model from the standard curve of each cytokine or chemokine.

### Phagocytosis assay

THP-1 monocytes were differentiated into mature macrophages by treatment with 100 nM PMA for 4 days before the experiment. J-Lat cells were exposed to NTP, incubated for 24 h, washed and labeled with Hoechst nuclear dye (ThermoFisher Scientific). THP-1 cells were labeled with wheat germ agglutinin (WGA) (Invitrogen, Cat. W32466). J-Lat and THP-1 cells were then co-cultured at a 1:1 E:T (effector:target) ratio for 1 h at 37°C, and supernatants from co-culture were saved for cytokine and chemokine measurements. Adherent, differentiated THP-1 cells were washed three times to remove non-internalized J-Lat cells. Co-culture at 4°C or pre-treatment of THP-1 cells with 100 μM cytochalasin D (Sigma-Aldrich, Cat. C8273) for 1 h prior to co-culture were used as negative controls for phagocytosis. The percentage of THP-1 cells that had engulfed labeled J-Lat cells was quantified using a flow cytometer (BD LSR Fortessa), and data was analyzed using the FlowJo software.

### MHC I peptide isolation and mass spectrometry

MHC I peptides were isolated from 5x10^6^ Jurkat or J-Lat cells that were either untreated or exposed for 15 s to NTP. Peptide isolation was accomplished using standard immunoaffinity purification as described previously [23]. In brief, ultra-low endotoxin azide free (LEAF)-purified anti-human HLA-ABC (clone W6/32 BioLegend) was coupled to cyanogen bromide (CNBr)-activated Sepharose (GE HealthCare), and cells were lysed. MHC-peptide complexes were isolated using 0.5 x 5 cm glass chromatography econo-column (BioRad) connected to a peristaltic pump (Ismatec). After elution, peptides were separated via filtration through a 3 kDa cut-off filter (Merck). Zip-Tip C18 (ThermoFisher) was used for desalting. Mass spectroscopy analysis was performed on an QExactive mass spectrometer with a nanoFlex source coupled to an UltiMate 3000 nano-liquid chromatography system (all Thermo Scientific) using 0.1% acetic acid (solvent A) and 95% acetonitrile with 0.1% acetic acid (solvent B). Samples were loaded onto a PepMap C18 precolumn (2 cm, 100 μm ID) and desalted for 4 min prior to being separated on a PepMap C18 column (15 cm, 75 μm ID, 3 nm particle size) using the following gradient: 2 min at 2% B, linear to 35% B in 64 min, linear to 50% B in 15 min, linear to 80% B in 5 min, followed by 10 min washing at 80% B and 10 min re-equilibration at initial conditions. The gradient was run at 200 nL/min, whereas washing and re-equilibration were performed at a flowrate of 500 nL/min. Separated peptides were analyzed by a Top10 DDA method using the following settings: MS1 spectrum acquisition between 300 and 1650 m/z with a resolution of 70,000, followed by 10 MS2 scans using 27.5 NCE, an isolation window of 2 m/z, and a resolution of 17,500. Dynamic exclusion was set to 30 s. The system was calibrated daily following standard procedures.

### MHC I peptide database search and ligand identification

Two peptide databases were generated using netMHCpan4.0 (23): one with HIV-1 proteins composed of 381 reviewed protein sequences (search query: HIV-1, organism: HIV-1, retrieved on May 7, 2020) and one with human proteins associated with different stages of the HIV-1 infectious cycle (including CD4, importin-7, and the DNA editing enzyme APOBEC3C) composed of 376 reviewed protein sequences (search query: HIV-1, organism: Human, retrieved on June 9, 2020) from the Uniprot.org database. Peptides with a length of 8, 9, 10, and 11 amino acids, and a cut-off rank of 2 for weak binders were predicted. For contaminants, peptides from the human proteome were predicted. Data processing was performed using Proteom Discoverer v2.4 (ThermoFisher). Peptide-Database, including HIV-peptides and human contaminants, was used for analysis by Sequest HT. Parameters included no enzyme digestion for peptides with a length of 7–12, precursor mass tolerance set to 5 ppm, and fragment mass tolerance set to 0.02 Da. Patterns of peptide display were summarized in a Venn diagram created with the online freeware tool (http://bioinformatics.psb.ugent.be/).

### Statistical analyses

Each experiment was performed in triplicate unless otherwise mentioned. Graphing and statistical analysis were performed using Prism 8.3 (GraphPad Software). Mean and standard errors were calculated using an unpaired Student’s t-test with Welch’s correction unless otherwise mentioned.

## Results

### NTP induces apoptotic programs in J-Lat cells and stimulates viral gene expression

In HIV-1 infection, the establishment of viral latency in CD4+ T cells presents a major barrier to the elimination of HIV-1-infected cells as these latently infected cells do not express HIV viral proteins and thus are not immunogenic. We thus investigated whether NTP application would induce expression of markers associated with immunogenicity in a model of latent HIV-1 infection: the Jurkat T-lymphocyte derived J-Lat (clone 10.6) cell line, which contains an integrated HIV-1 genome.

**Fig 1:**
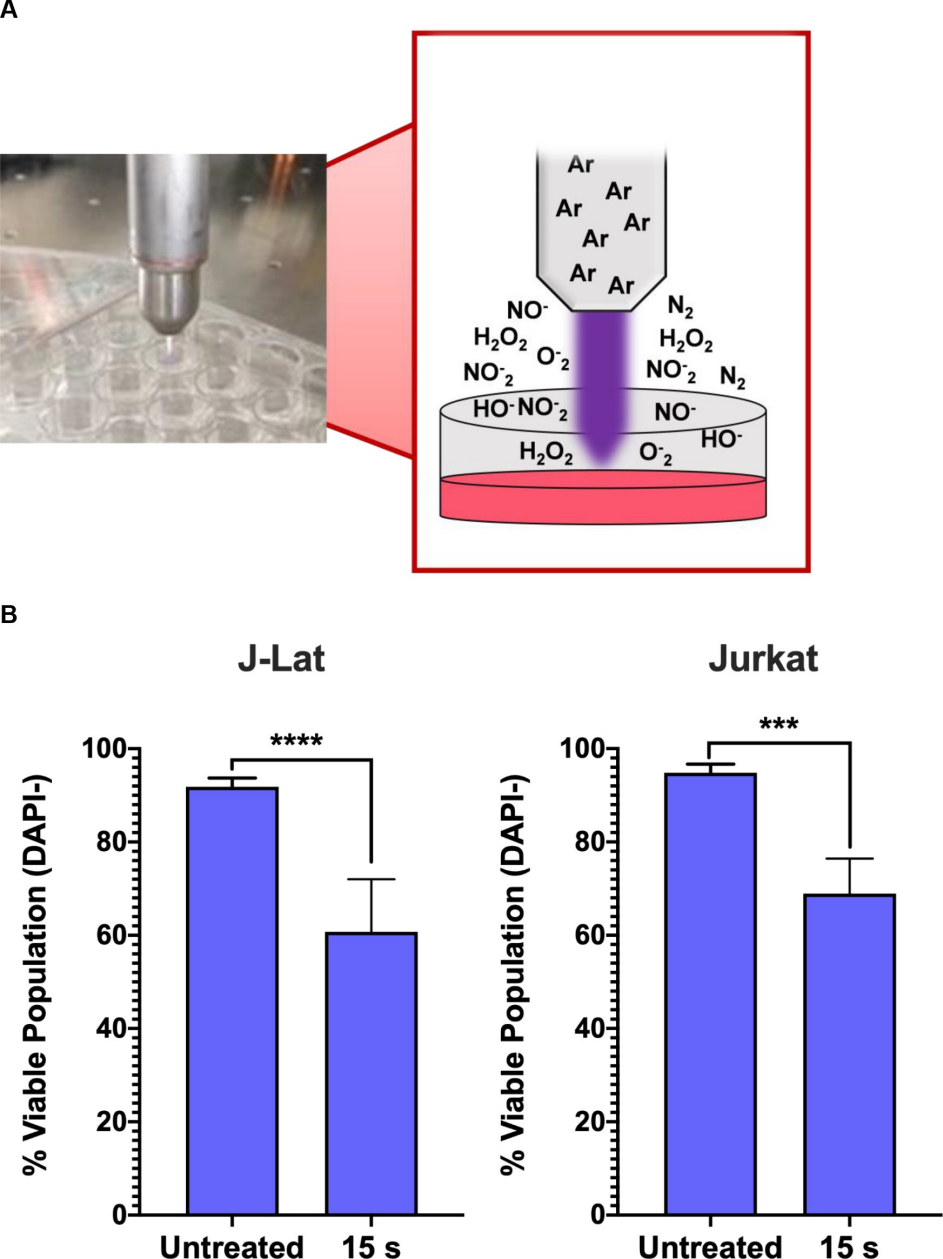
NTP device and experimental set-up. (A) NTP was generated using a kINPen plasma device operating with an argon feed gas. Cells were seeded at 4x10^5^ cells/mL into 24-well plates with 500 μL aliquoted into each well. For each NTP exposure, the pen was positioned directly above each well to allow for direct application to each sample. (B) NTP exposure of J-Lat cells at 15 s resulted in a decrease of the viable population similar to the parent Jurkat cell line, as measured via flow cytometry. Data are presented as mean ± SD of at least 2 independent experiments. p-values were calculated using unpaired Student’s t-test with Welch’s correction. ****p<0.0001.

In our experimental set-up, we used a kINPen device, which generates NTP composed of various RONS that accumulate in concentration with increasing exposure duration. NTP was applied directly to each well of cells in a tissue culture plate (Fig 1A). We tested a dose range of 5–20 s during which NTP may induce low to high cytotoxicity as a result of NTP-induced oxidative stress, based on the T-lymphocyte sensitivity to NTP reported in our previous studies [24,25]. In this range, the 15 s exposure resulted in a 30% decrease in the viable population of J-Lat cells 24 h post NTP exposure. No appreciable difference in the loss of viability after NTP exposure was observed between these cells and the parent Jurkat cell line (Fig 1B), suggesting that there was negligible impact of the integrated provirus on NTP-induced cell death. Prior studies have demonstrated that NTP treatment of various mammalian cells, including Jurkat cells and primary T lymphocytes, commonly results in cytotoxicity that is dose-dependent [24,26,27]. This is a shared feature among the different devices used for NTP generation. Furthermore, it was demonstrated that the cytotoxicity induced by NTP is primarily apoptotic death [28–30].

**Fig 2:**
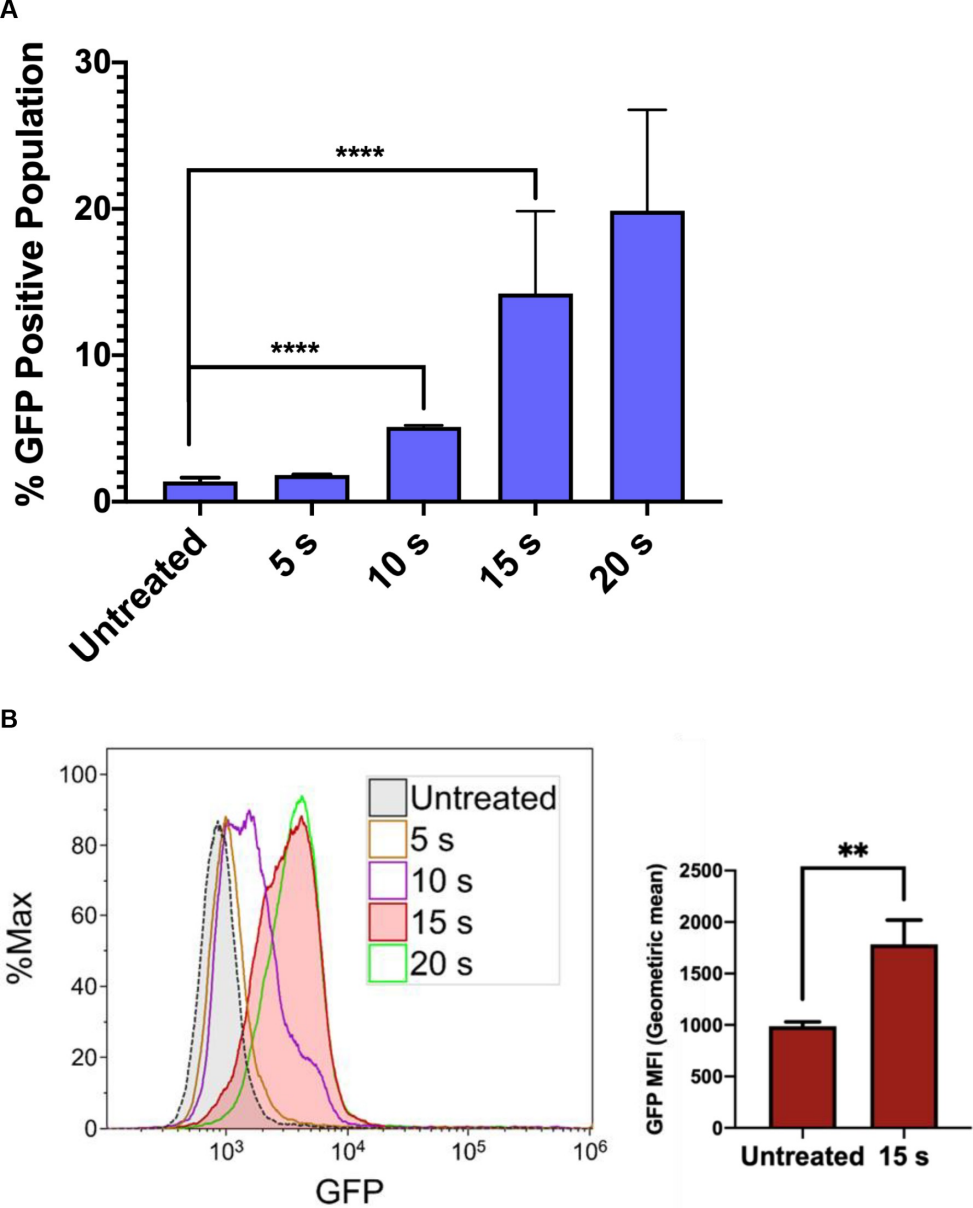
NTP induces a dose-dependent increase in HIV-1 gene expression in J-Lat cells. J-Lat cells were exposed to NTP for 5–20 s, and GFP production was measured via flow cytometry 24 h later. (A and B) GFP expression was stimulated in NTP-exposed cells in a dose-dependent manner, resulting in increases in the percentage of GFP-positive cells in the population as well as GFP intensity. Data are presented as mean ± SD for percent positive and mean ± SEM for MFI, from four independent experiments for untreated and 15 s treated samples. p-values were calculated using unpaired Student’s t-test with Welch’s correction. **p<0.01, ****p<0.0001.

We next investigated the influence of NTP exposure on the latent state of J-Lat cells, measuring viral reactivation through the expression of the GFP reporter carried by the cells. Incubation of J-Lat cells with 100 nM phorbol 12-myristate 13-acetate (PMA) (as a positive control) resulted in a 70% or greater GFP positive cell population (S1 Fig). Surprisingly, we observed that NTP exposure for 15 s increased the number of GFP-positive J-Lat cells by over 10-fold (1.4% vs. 14.2%, p<0.0001) and increased GFP intensity in individual cells (1.8-fold, p = 0.0069) compared to untreated cells. This result suggests that NTP acts as an inducer of HIV-1 gene expression and that viral genes may also be highly expressed in NTP-exposed cells. Moreover, this effect was observed to be dose-dependent, suggesting that HIV-1 reactivation can be modulated (Fig 2A and 2B). These results indicate that NTP has the potential to enhance the immunogenicity of latently infected cells by reactivating viral gene expression and facilitating better recognition by, and stimulation of, innate or adaptive immune cells. Because immunogenicity requires more than just antigen expression, we further investigated the ability of NTP to modulate mediators and other markers associated with immunogenicity in this T-cell line model of latent HIV-1 infection.

### NTP exposure increases the display and release of multiple DAMPs associated with promotion of APC function

**Fig 3:**
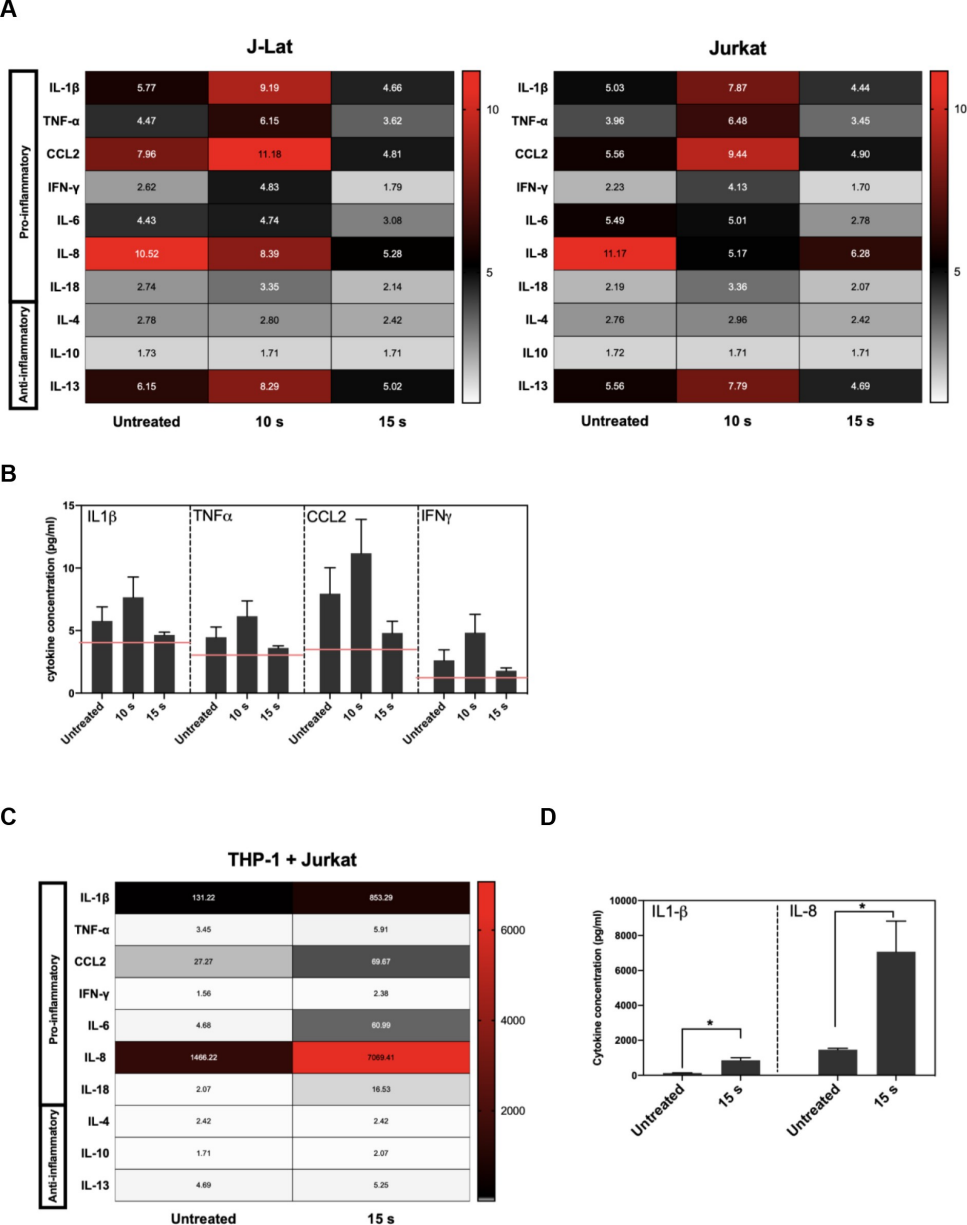
Pro-inflammatory cytokines and chemokines are released in NTP-exposed J-Lat cells. J-Lat cells were exposed to NTP, and supernatants from cultures were collected 24 h later for detection of pro-inflammatory cytokine release. (A and B) The release of several pro-inflammatory cytokines occurred for both J-Lat cells and Jurkat cells 24 h following NTP exposure, with a moderate increase in released IL-1β, TNF-α, CCL2, and IFN-γ from J-Lat cells. (C and D) Jurkat cells that were 15 s NTP exposed and incubated for 24 h were co-cultured with THP-1 cells for 1 h. Supernatant collected from these co-cultures were assayed for pro-inflammatory cytokine release and found to be most abundant in IL-1β and IL-8 (Mann-Whitney *U* test, *p < .05). Values in heat map correspond to concentrations in pg/mL, and red lines indicate limit of detection (LOD). Data are represented as mean ± SEM from at least two independent experiments with at least four replicates. p-values: **p<0.01, ***p<0.001, ****p<0.0001.

Effective APC function in response to infected cells relies on the display of DAMPs that promote uptake as well as the release of pro-inflammatory cytokines driving maturation and subsequent cross-presentation of dead cell-derived antigens to CD8+ T cells. Since APC function may be strongly modulated by the cytokine and chemokine milieu established by NTP-exposed cells, the release of pro-inflammatory cytokines and chemokines from J-Lat cells at 24 h was assessed after a 15 s NTP exposure. One chemokine that has been used as an adjuvant in vaccination studies is HMGB1, a nuclear resident protein that is released in response to certain kinds of cellular stress [31–34]. Release of this pro-inflammatory chemokine from J-Lat cells was stimulated by NTP application. Furthermore, HMGB1 release and GFP reporter expression stimulated by NTP exposure were both dose-dependent (S2 Fig). Among the other released chemokines and cytokines assayed, there were discernable increases, albeit at low concentrations, in released IL-1β, TNF-α, IFN-γ, and CCL2, relative to untreated cells. Unexpectedly, the release of cytokines was only evident at a lower NTP dose (10 s). Considering NTP induces a dose-dependent cytotoxicity resulting in cell loss, this may be due to altered cell-to-cell cytokine signaling. Importantly, while the overall pro-inflammatory cytokine profile was the same for NTP-exposed J-Lat cells and the parent Jurkat cell line, NTP-exposed J-Lat cells released a higher concentration of pro-inflammatory cytokines relative to NTP-exposed Jurkat cells, particularly IL-1β and CCL2 (Fig 3A and 3B). This may be a consequence of the added effect of viral gene expression on stimulation of innate immune signaling cascades.

To better understand the influence of NTP-mediated effect on APC function in the absence of viral gene expression, we quantified the cytokines in supernatants of untreated THP-1 cells co-cultured with Jurkat cells that were incubated for 24 h after being exposed to NTP. The release of pro-inflammatory cytokines and chemokines was amplified 24 h following co-culture (Fig 3C). In addition to much greater concentrations of IL-1β and CCL2, an increase in soluble IL-8 was noted in the co-culture supernatants (Fig 3C and 3D). The source of IL-8 release was likely THP-1 cells, as macrophages release IL-8 following encounters with foreign antigens in order to recruit neutrophils that contribute to more efficient phagocytosis necessary to clear viral infection [35–37]. In comparison, release of anti-inflammatory cytokines such as IL-10 was not notably increased in these co-cultures, further supporting the conclusion that NTP exposure of T lymphocytes may promote rather than inhibit APC functions.

NTP-mediated cytotoxicity has often been associated with translocation of intracellular proteins to the cell surface, where they serve as DAMPs that promote uptake or phagocytosis of infected or tumor cells. Some of these proteins, including calreticulin (CRT), have been shown to enhance immunogenicity in animal models due to promotion of phagocytosis of dead cells in an immunosuppressive environment [16,19,38,39]. Likewise, the presence of the heat shock proteins HSP70 and HSP90 has also been shown to greatly enhance immunogenicity [39,40].

**Fig 4:**
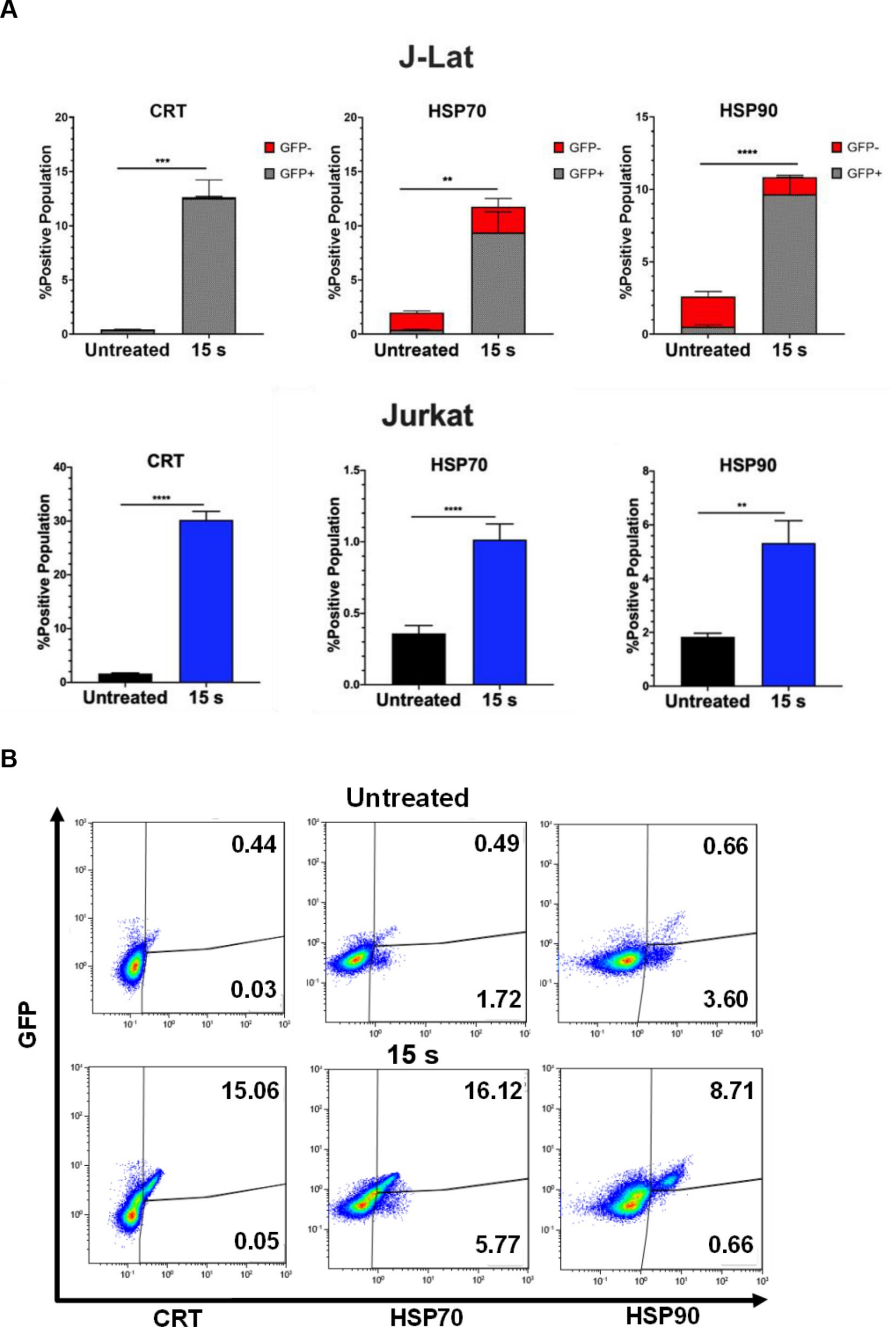
NTP increases cell surface display of pro-phagocytic DAMPs on J-Lat cells and Jurkat cells. J-Lat cells and Jurkat cells exposed to 15 s of NTP were incubated for 24 h and assayed for pro-phagocytic DAMPs on the cell surface using flow cytometry. (A) The percentage of the NTP-exposed cell population displaying the pro-phagocytic DAMPs calreticulin (CRT), HSP70, and HSP90 on J-Lat cells and Jurkat cells was increased compared to the untreated control for both cell lines. (B) The majority of NTP-exposed J-Lat cells displaying pro-phagocytic DAMPs were also GFP-positive. Data are presented as mean ± SEM from at least three independent experiments with three replicates. p-values: **p<0.01, ***p<0.001, ****p<0.0001.

Therefore, we quantified pro-phagocytic DAMPs CRT, HSP70, and HSP90 on cells exposed to NTP for 15 s and then incubated for 24 h. In this regard, the presence of all three DAMPs was shown to be increased on the surface of J-Lat cells, with an over 28-fold increase in CRT (p = 0.0001), 5-fold increase in HSP70 (p = 0.0081), and 4-fold increase in HSP90 (p<0.0001) marker-positive cells relative to cells not exposed to NTP. Jurkat cells, in contrast, only had an 18-fold increase for CRT (p<0.0001), 2.8-fold for HSP70 (p = 0.0001), and 1.8-fold for HSP90 (p = 0.0011) (Fig 4A). This larger increase in display of pro-phagocytic DAMPs may reflect an additive outcome of cell stress induced by NTP and by viral gene expression. Further examination of this population of cells showed that the majority of cells displaying pro-phagocytic markers were GFP-positive. This suggests that NTP would promote preferential APC targeting and elimination of cells that simultaneously express DAMPs and HIV-1 genes, by APCs (Fig 4B). The majority of GFP-positive cells were also positive for at least one of these pro-phagocytic DAMPs (S3 Fig). Together, these results provide evidence that NTP may be used to modulate both the display of immunogenic DAMPs and release of pro-inflammatory cytokines and chemokines to promote APC functions, including recruitment, phagocytosis, and maturation.

### NTP-exposed J-Lat cells are efficiently phagocytosed by THP-1 macrophages

**Fig 5:**
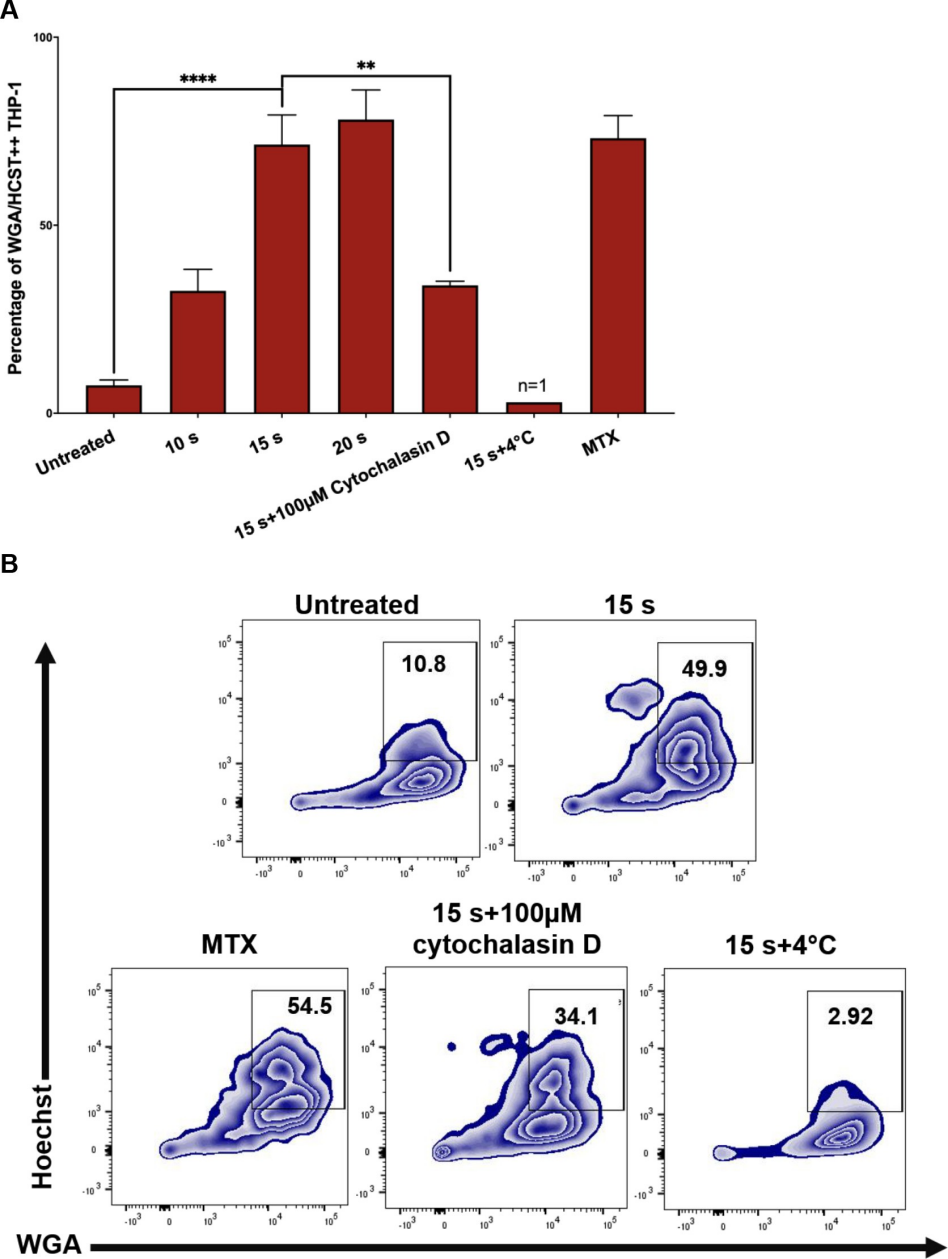
NTP exposed J-Lat cells are efficiently phagocytosed by THP-1 macrophages. Phagocytosis of J-Lat cells was assessed using *in vitro* co-cultures with macrophages. (A and B) J-Lat cells were incubated for 24 h, then labeled with Hoechst nuclear stain prior to the phagocytosis assay. Macrophages were differentiated by stimulating THP-1 monocytes with 100 nM PMA for 4 days, then labeled with wheat germ agglutinin (WGA) and co-cultured at a 1:1 E:T ratio with the Hoechst labeled J-Lat cells for 1 h. Co-cultures were examined for phagocytosis via flow cytometry, by evaluating the percentage of THP-1 cells that were double-positive for WGA and Hoechst, corresponding to THP-1 cells that had engulfed J-Lat cells. Uptake of NTP-exposed J-Lat cells (15 s exposure) by THP-1 cells 1 h post co-culture was greatly enhanced compared to uptake of untreated cells, and phagocytosis was observed to be dose-dependent as co-culture with J-Lat cells exposed to increasing NTP doses yielded increased engulfment of J-Lat cells by the THP-1 macrophages. J-Lat cells treated with 250 nM mitoxantrone (MTX) were used as a positive control. As a negative control, THP-1 cells were treated with 100 nM cytochalasin D for 30 min prior to co-culture with NTP-exposed J-Lat cells (15 s), or co-cultured at 4°C for 1 h. Both control approaches yielded reduced J-Lat cell uptake (n = 1). Data are presented as mean ± SEM from two independent experiments with at least four replicates. p-values: **p<0.01, ****p<0.0001.

The display of CRT, HSP70, and HSP90 on J-Lat cells along with release of pro-inflammatory cytokines following NTP exposure should make them good candidates for efficient phagocytic uptake by APCs. This possibility was evaluated in a phagocytosis assay, in which THP-1 macrophages pre-labeled with wheat germ agglutinin (WGA) were co-cultured with NTP exposed J-Lat cells that had been pre-labeled with Hoechst nuclear stain. THP-1 and J-Lat cells were co-cultured at a 1:1 ratio for 1 h and then assayed for phagocytosis using flow cytometry. Phagocytosis completion was calculated as the percentage of THP-1 cells that were double-positive for both cellular dyes, indicative of macrophages that contained internalized J-Lat cells. Doublet exclusion was used to gate out J-Lat cells that were attached to macrophages rather than internalized. NTP exposure promoted increased phagocytosis of J-Lat cells by THP-1 macrophages, resulting in over a 9-fold increase in percent phagocytosis for 15 s exposed cells as compared to untreated cells (p<0.0001) (Fig 5A). Enhancement of phagocytosis was observed to be dose-dependent, as longer exposures of J-Lat cells to NTP resulted in greater levels of J-Lat cell engulfment. Phagocytosis of NTP-exposed J-Lat cells was reduced 4.5-fold (p = 0.0014) by a 30 min pre-treatment of differentiated THP-1 cells with 100 μM cytochalasin D, supporting the conclusion that J-Lat cells were engulfed by THP-1. Similarly, effective negation of phagocytosis by co-culture at 4°C (n = 1) validates the conclusion that double-positive cells were indicative of phagocytosed J-Lat cells, since the lower incubation temperature would reduce macrophage cell membrane fluidity and impede phagocytosis (Fig 5B).

### Surface expression of molecules associated with antigen presentation is modulated by NTP exposure

In ART suppressed PLWH, weak cytotoxic CD8+ T-cell (CTL) responses and reduced recognition of HIV-1-infected cells are barriers to clearance of infected cells [41–43]. The downregulation of MHC I on infected cells is a major contributor to poor recognition and killing by CTLs. Decreased surface expression of other molecules such as the costimulatory CD28 has also been associated with disease progression [11]. In response to viral protein production, cells upregulate MHC I molecules that display virus-derived peptides for recognition and elimination of the infected cell by CD8+ T cells. Thus, in addition to assaying for the potential of NTP to modulate APC function, the effect of NTP on the display of surface markers necessary for recognition and targeting by CD8+ T-cells or other immune cells was also assessed.

**Fig 6:**
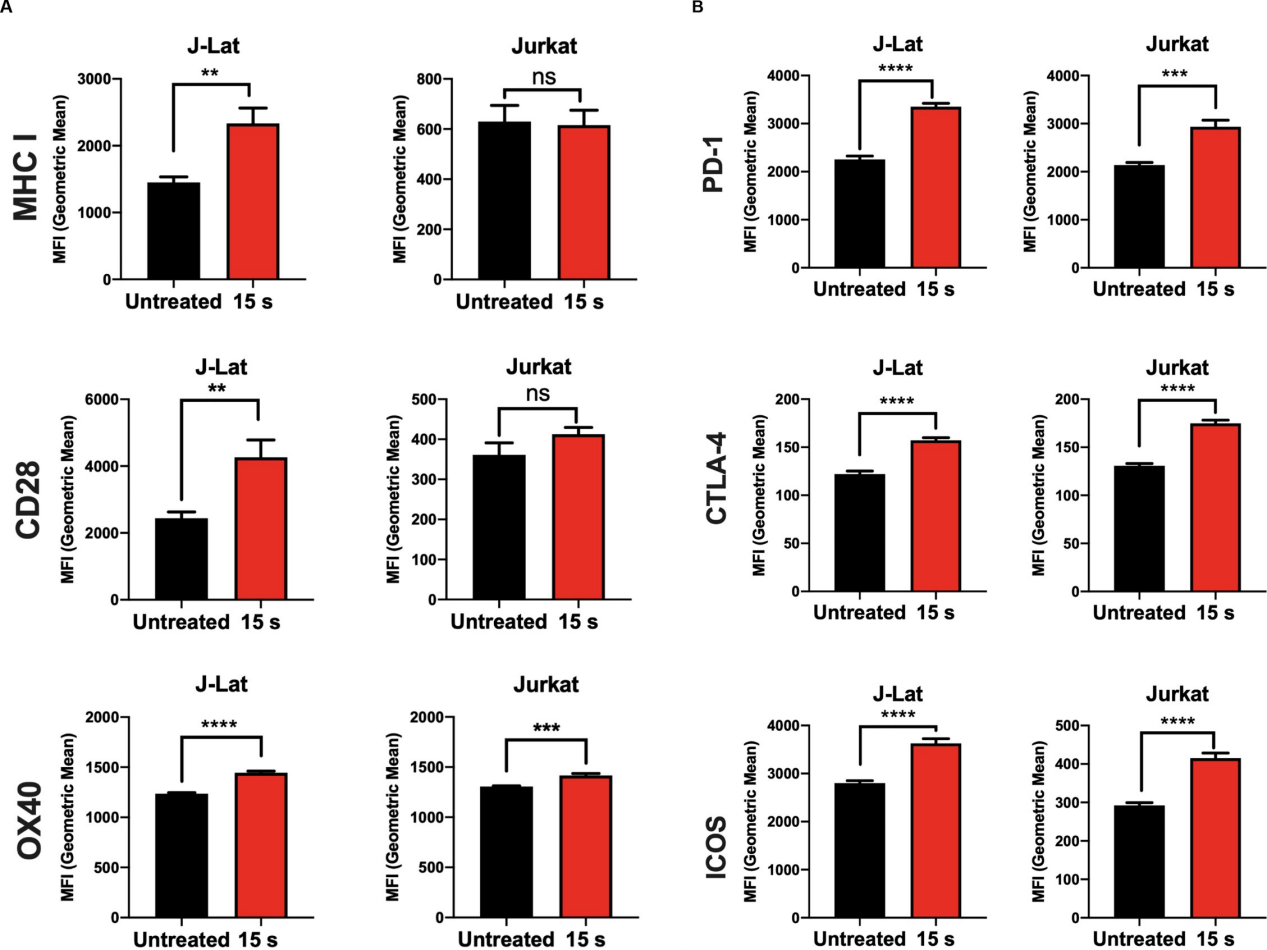
MHC I and costimulatory molecule expression is modulated in NTP-exposed cells. Cell surface expression of molecules associated with antigen presentation was assayed on Jurkat and J-Lat cells via flow cytometry 48 h following NTP exposure. (A and B) NTP modulated expression of MHC I and costimulatory CD28 and OX40 primarily on J-Lat cells. (C) Surface expression of inhibitory PD-1, CTLA-4, and ICOS was considerably increased for both J-Lat and Jurkat cells. Data are presented as mean ± SEM from at least three independent experiments with three replicates. p-values: **p<0.01, ***p<0.001, ****p<0.0001.

An increase in MHC I on the cell surface following NTP exposure has been reported previously in melanoma cells as early as 24 h post-exposure [21]. Upregulation of costimulatory molecules following NTP exposure, however, has not been reported, despite the importance of these molecules in stabilizing antigen presentation. In this regard, the surface expression of MHC I as well as costimulatory molecules CD28, OX40, and CD40L that are known to enhance the stability of antigen presentation was measured. In contrast to the previous study, we found that upregulation of MHC I was more evident at 48 h as opposed to 24 h post-NTP exposure and was accompanied by moderately increased surface expression of CD28 and OX40, but not CD40L. However, Jurkat cells did not have a similar change in expression of these surface molecules (Fig 6A).

The effector function of T cells as well as antigenicity can be regulated by the inhibitory checkpoint molecules. In PLWH, increased expression of the checkpoint markers programmed cell death protein 1 (PD-1), cytotoxic T-lymphocyte-associated protein 4 (CTLA-4), and inducible T-cell costimulatory (ICOS) on HIV-1 infected CD4+ T cells has been reported and may be the reason for poor clinical outcomes in studies of novel immunotherapies [44–52]. To this point, the effect of NTP on exposure of these inhibitory checkpoint markers was also assessed. While NTP exposure of J-Lat cells induced modest but significant increases (compared to untreated cells) in the cell surface display of MHC I (1.6-fold, p = 0.0040) and the costimulatory molecules CD28 (1.7-fold, p = 0.0078) and OX40 (1.2-fold, p<0.0001) (Fig 6A), NTP also caused significant increases in PD-1 (2.4-fold, p<0.0001), CTLA-4 (1.3-fold, p<0.0001), and ICOS (1.3-fold, p<0.0001) (Fig 6B). This effect was also observed in Jurkat cells, in which there were increases in the surface expression of PD-1 (1.4-fold, p = 0.0005), CTLA-4 (1.4, p<0.0001), and ICOS (1.4-fold, p<0.0001). However, there was no significant change in surface MHC I nor CD28 and only a small increase in OX40 (1.1-fold, p = 0.0008) (Fig 6B), suggesting that increases in these markers may be attributed to direct effects of NTP exposure. The implications of the simultaneous cell surface display of markers that promote and those that inhibit efficient antigen presentation as a prelude to an effective CD8+ T cell response have yet to be determined.

### NTP exposure alters the MHC I peptide pool

**Fig 7:**
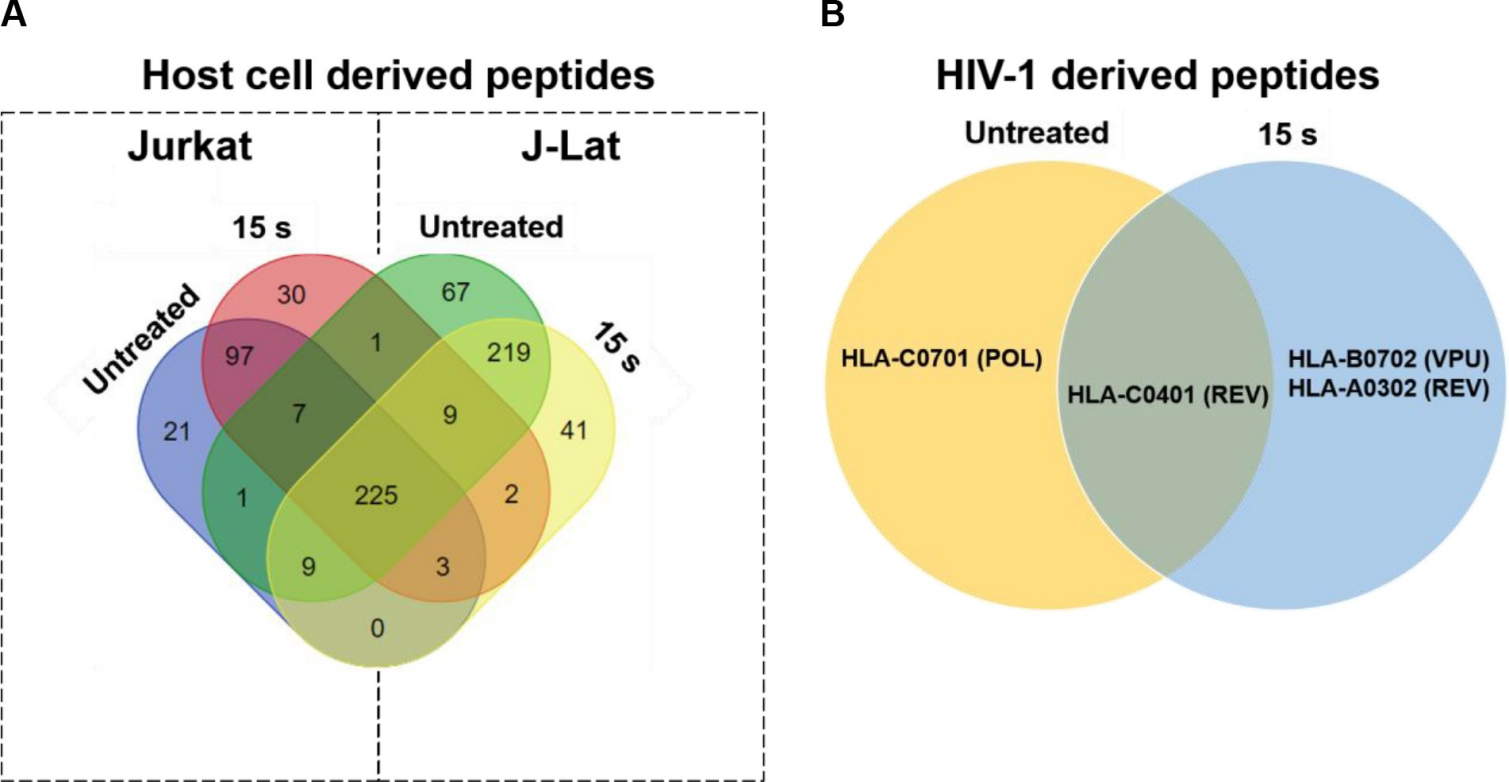
NTP exposure alters the display of HIV-1-derived peptides on MHC I. Jurkat and J-Lat cells (each n = 2) were exposed to NTP and then collected 24 h later for purification of MHC I-peptide complexes. (A and B) Peptides were analyzed via mass spectrometry and then checked for matches to several human proteins involved in HIV-1 infection or HIV-1 proteins. Abbreviations for HIV-1 proteins: REV = regulator of expression of virion proteins, POL = DNA polymerase, VPU = viral protein U.

Proper targeting of HIV-1-infected cells by CD8+ T cells requires presentation of immunogenic peptides in the cleft of MHC I on the infected cell. Increasing the breadth and magnitude of HIV-1 antigen presentation on an HIV-1-infected cell has the potential to stimulate more robust CTL responses, particularly if they are both augmented. An increase in the diversity of the peptides presented on infected cells can also enhance CTL recognition and targeting in individuals with HLA types that are associated with disease progression due to unstable binding of the MHC-peptide complex with the T cell receptor (TCR) of CTLs [53,54]. We have shown an increase in both, viral gene expression in J-Lat cells, and surface expression of MHC I in response to NTP exposure. To test the hypothesis that activation of viral expression by NTP would be followed by increased antigen processing of viral proteins, the change in the array of peptides presented on NTP-exposed cells was examined. We assayed for peptides only derived from HIV-1 proteins, and then assessed the influence on presentation of host cell protein-derived peptides, focusing on those that play a role in the HIV-1 infectious cycle, including cellular proteins important for viral entry and DNA integration. Mass spectrometry analysis demonstrated that over 225 MHC I peptides of host cell (human protein) origin were detected in mock-exposed or NTP-exposed J-Lat cells, which were derived from proteins that are associated with the HIV-1 infectious cycle. However, these peptides were also detected in Jurkat samples, NTP treated or not (Fig 7A). For NTP-exposed J-Lat cells, 41 unique peptides were detected which were not found in untreated cells, while Jurkat cells presented 30 altered peptides (Table 1). Analysis of HIV-1 proteins showed a much lower yield of detected peptides in these samples (Fig 7B). In NTP-treated J-Lat cells, a single peptide derived from the HIV-1 regulatory protein Rev, which was associated with HLA-A0302, and one peptide derived from the accessory protein Vpu, associated with HLA-B0702 were detected. While the overall yield of HIV-1 peptides was too low to confirm enhanced depth and breadth of antigens displayed on NTP-treated cells, a comparison of unique peptides from viral and human origin between treated and untreated samples is suggestive of novel peptides displayed with NTP application.

**Table 1:** List of unique peptides presented on NTP-treated (A) J-Lat and (B) Jurkat cells.

**A. J-Lat (15 s)**				
**Sequence**	**Accession**	**Master Protein**	**Binding Affinity**	**HLA-Allele**
MALLTAETF	Q6NTF7	ABC3H	0.15	HLA-B3501
RVVKRFILHA	P61221	ABCE1	1.34	HLA-A0301
DLYRSGYDY	Q9ULX6	AKP	0.77	HLA-B3501
ALLPAATQGK	P01730	CD4	0.3	HLA-A0301
RAAEAARAA	Q14004	CDK	1.51	HLA-B0702
RQKSNEVW	Q9P126	CLC1B	1.1	HLA-A0301
LKYPHVAERVY	P20813	CP2B6	1.69	HLA-B3501
GPAGSGRSG	Q9Y5B0	CTD	1.63	HLA-B0702
TTVILILAF	P61073	CXC	1.63	HLA-B3501
QAKRMTWG	O00574	CXCR6	1.96	HLA-A0301
SWDTNDANV	Q9UGM3	DMB	0.13	HLA-C0401
GLRPRFPVLL	P55265	DSR	1.55	HLA-C0701
ARIERITRRF	P55199	ELL	0.2	HLA-C0701
RALKLPNLV	P06241	FYN	1.79	HLA-C0701
RQRPIPLSM	Q8WUF5	IASPP	0.56	HLA-C0401
SPRKARRARL	Q8WUF5	IASPP	0.05	HLA-B0702
SRKIQIRNI	Q9NZI8	IF2B1	0.36	HLA-C0701
VEAPPKILV	P53990	IST1	1.77	HLA-C0401
APGNTHGSF	P06239	LCK	0.06	HLA-B0702
MLSLDASAM	Q6UWE0	LRSM1	1.05	HLA-B3501
FGDIFLHL	Q8WX92	NELFB	1.45	HLA-C0401
IGNMARPLL	P28074	PSB	1.66	HLA-C0401
MLKGCVVGT	P39023	RL3	0.55	HLA-A0301
AMGKQAMGVY	P30876	RPB2	1.8	HLA-A0301
GVSDFCET	P30876	RPB2	0.89	HLA-A0301
RLRAYAK	P19387	RPB3	1.03	HLA-A0301
ILYSTDP	Q9Y3Z3	SAM	1.51	HLA-A0301
TPQFQRLRY	Q9Y3Z3	SAM	1.39	HLA-B0702
FPVPPGHL	Q68D06	SLN	0.25	HLA-B0702
RVSNFKPGVY	P63272	SPT4H	1.05	HLA-A0301
FRSGVEIIRM	O95630	STA	0.32	HLA-C0701
KFWENYILIM	Q13395	TARB1	1.64	HLA-C0401
NAYVKSIVQEY	Q13395	TARB1	1.2	HLA-B3501
KLLDVVHPAA	Q99832	TCP	0.22	HLA-A0301
QRLMLRRVAL	O15455	TLR3	1.82	HLA-C0701
VPYLAYIL	Q92973	TNP	0.55	HLA-B0702
YILDTLVFAF	Q92973	TNPO1	1.78	HLA-C0401
SRPISASY	Q99816	TS101	1.03	HLA-C0701
HPKRPMHI	Q969X6	UTP	1.27	HLA-B0702
MPAPLYSPT	P63131	VPK7	0.75	HLA-B0702
MLKKHIRTH	P15822	ZEP	1.75	HLA-A0301
**B. Jurkat (15 s)**				
**Sequence**	**Accession**	**Master Protein**	**Binding Affinity**	**HLA-Allele**
TLTKIALRY	P53618	COPB	0.33	HLA-A0301
QRENSPAAF	Q9Y5B0	CTDP1	1.9	HLA-C0401
LAFISLDRY	P61073	CXCR4	0.34	HLA-B3501
GPRPPKMAR	Q08211	DHX	1.28	HLA-B0702
GIGKNQPRN	Q9UPY3	DICER	1.33	HLA-A0301
AIVCWKPG	O75530	EED	0.83	HLA-A0301
GATDAVPSR	P55199	ELL	0.77	HLA-A0301
FLEAFSPVYKY	P19447	ERCC3	2	HLA-B3501
GPADPAVGDM	Q6DN72	FCR	0.38	HLA-B0702
PRPRPLWATVL	P05106	ITB	0.56	HLA-B0702
CPDGFQLVA	P01130	LDL	1.45	HLA-B3501
VIAAPSMVAM	Q09161	NCBP1	1.87	HLA-B0702
RASGVSAAA	Q8WX92	NEL	1.42	HLA-B0702
SSPVKTTSQA	P19838	NFK	1.53	HLA-B0702
HPAPGFPGG	Q5VVQ6	OTU1	1.41	HLA-B3501
VPVYPHNPWF	Q01804	OTUD4	0.75	HLA-B3501
LPHSTVKTFTL	P63135	POK7	0.88	HLA-B0702
QPVKVFRLL	P63128	POK9	0.68	HLA-B3501
FRSNFGYNIPL	P25788	PSA	1.74	HLA-C0701
LKPKSFGMYL	P28074	PSB	0.33	HLA-B0702
RSLLLQKKLH	O15514	RPB4	1.46	HLA-A0301
IYDEIQQEM	Q01826	SAT	0.21	HLA-C0401
AAAAGMGPR	Q7Z6J0	SH3R1	1.36	HLA-A0301
LIFQKDYLEY	Q7Z7L1	SLN11	1.66	HLA-B3501
RVMEIVDADE	Q9Y5B9	SP16H	1.32	HLA-A0301
TPAMYNTDQF	O00267	SPT5H	0.43	HLA-B3501
MTTKDLLK	P35269	T2FA	0.42	HLA-A0301
HVIKPVLP	Q13395	TARB1	0.02	HLA-A0301
EQLMGAVVM	Q9UN37	VPS4A	1.2	HLA-B3501
DATKEAVAA	O95365	ZBT7A	1.98	HLA-B3501

## Discussion

ART has been very successful in reducing the burden of HIV infection and prolonging the lives of HIV-1 infected subjects, but is associated with serious side effects including cardiovascular and kidney diseases [55,56]. Furthermore, it is not curative, allowing the virus to persist in the form of latent reservoirs. The challenge in eliminating the latent reservoir stems from a combination of low viral protein expression in HIV-1 infected cells and impaired innate and adaptive immune responses even when latently infected cells are forced to express HIV-1 protein. While several groups are exploring the potential of latency reversal agents (LRAs) to eliminate the HIV-1 reservoirs *in vivo* [57–59], there is an unmet need for therapeutic approaches that can resolve the immune dysfunction in PLWH and induce a robust CD8+ T lymphocyte response necessary to control infection when latency is reversed.

Herein, we describe a novel NTP-based immunotherapeutic approach that induces virus gene expression and other cellular responses in latently infected cells that improves their engagement with APCs to potentially enhance the downstream CD8+ T lymphocyte recognition and killing of infected cells. To our knowledge, this is the first report of NTP-mediated enhancement of immunogenicity in HIV-1 infection. We show that exposure to NTP stimulates viral gene expression in the J-Lat cell line, release of pro-inflammatory cytokines, increased display of pro-phagocytic DAMPs and phagocytosis by THP-1-derived macrophages. These are accompanied with the increased expression of surface molecules associated with antigen presentation. Furthermore, an altered array of peptides presented on NTP exposed CD4+ T cells is demonstrated. Thus, NTP has the potential to stimulate both the innate and adaptive immune function relevant for overcoming various dysfunctions of the immune response for control of HIV-1.

A limited number of studies have reported the *in vitro* effects of NTP on HIV-1 infectivity and infection. Volotskova and colleagues showed that treatment of macrophages with NTP prior to HIV-1 infection prevented viral entry by altering cellular receptors and subsequently reduced infection [60]. Another study evaluated the effect of NTP on HIV-1 infection in HeLa-based reporter cells by treating HIV-1 virions with NTP prior to infection or exposing cells after infection. In both cases, decreases in HIV-1 replication and titer were observed [61]. Both studies were focused on evaluating the antiviral potential of NTP and not the immunomodulatory potential of NTP in the context of HIV-1 infection.

The multifaceted nature of NTP-increased immunogenic markers in J-Lat cells is indicated by the release or display of various DAMPs that promote APC activity. The released pro-inflammatory cytokines HMGB1, CCL2, and IL-1β have important roles in monocyte recruitment and differentiation of APCs [62–67]. Additionally, we show that NTP increases the display of CRT, HSP70, and HSP90, correlating with effective phagocytosis of NTP-exposed cells (Fig 4). While these results have suggested that NTP can modulate the expression of immunogenic markers, we suspect that the effects observed may be even more pronounced in cells from patients with HIV-1 infection. This is in part because HIV-1, as with several other viral infections, is known to increase oxidative stress of CD4+ T cells, and this may be added to the stress of NTP application, especially with increased Vpr expression [68–72].

Many of these DAMPs are documented markers of immunogenic cell death (ICD), and their chemical and physical inducers are being explored as *ex vivo* cancer therapies to enhance APC function for protection against cancer in whole-cell vaccination strategies [73–75]. Following the logic from cancer studies, it is anticipated that the *in vivo* administration of HIV-1 infected cells exposed *ex vivo* to NTP will enhance APC function. To investigate the systemic expansion of HIV-1-specific cytotoxic CD8+ T cells following administration of NTP-exposed, HIV-1-infected cells, future studies will employ an animal model of infection and treatment. Our observation that the majority of NTP-exposed J-Lat cells displaying pro-phagocytic markers are GFP-positive also suggests that APCs target infected rather than uninfected NTP-exposed cells. The impact of phagocytosis on subsequent cross-presentation of viral antigens require HLA-matched cell sets. Thus, these will be assessed using APCs differentiated from HIV-1-infected patient monocytes and paired CD8+ T cells, that will more closely reflect the innate immune response in PLWH.

The accompanying increase in surface expression of MHC I on J-Lat cells in our study suggests that NTP-exposed CD4+ T cells, when administered to subjects, will likely be targeted *in vivo* by memory CD8+ T cells. Another important determinant of the killing efficiency of CTLs is the antigenicity of the peptides being presented; the presentation of novel antigens provides an opportunity for a new, more robust systemic response. In our experiments, we show that J-Lat cells present a small, albeit different, array of peptides after NTP exposure, thus providing potential new antigens for CTLs. However, the observed increase in PD-1, ICOS, and CTLA-4 may serve to modulate the response of CD8+ T cells or may simply be a mechanism of regulating the immune responses. To investigate this, we plan to assess the efficiency of CTL-mediated killing of NTP-exposed cells and examine the impact of NTP-stimulated presentation of new antiviral antigens on CTL function.

The NTP-mediated latency reversal of HIV-1 infection is another novel observation of this study and suggests a role for dominant RONS (or a combination of RONS) delivered by or induced by NTP in the reversal of the quiescent state of CD4+ T cells. The described mechanisms of reversing latency in resting CD4+ T cells include direct inhibition of transcriptional silencers (histone deacetylases or Bromo- and Extra-Terminal domain (BET) family proteins), and stimulation of the protein kinase C (PKC) intermediate in the T-cell activation pathway [59]. Considering NTP induces oxidative stress in cells and ROS have been reported to act as second messengers that can stimulate NF-κB [76,77], the mechanism whereby viral gene expression is stimulated by NTP application is likely more upstream akin to that of PKC agonists but has yet to be elucidated. Furthermore, the molecular mechanism by which NTP modulates the antigen presentation pathway is not known. Other more robust latency reversal agents have been reported to alter antigen processing by affecting the breadth and production of HIV-1 peptides presented on MHC I [78].

While the immunomodulatory effects of NTP in our model of HIV latency are encouraging, we recognize that the leukemic origin of J-Lat cells introduces constraints on the conclusions we can draw. Additionally, the integrated provirus in this cell line has been further modified so that Nef, a protein that plays an important role in virus replication, is replaced by the GFP reporter gene [79]. Nef has been shown to sequester MHC I intracellularly, thus protecting the infected cell from CD8+ T-cell surveillance and killing [80]. Therefore, it is difficult to evaluate if NTP can reverse the effect of Nef on MHC I trafficking to the cell surface in these cells. Assessment of the NTP-mediated increase in MHC I expression on CD4+ T cells from PLWH would better address this.

Collectively, our data demonstrate the simultaneous increase of multiple markers of enhanced immunogenicity, with potential implications in overcoming different deficiencies of the immune system that limit HIV-1 control in PLWH. We believe that these effects will be more pronounced in cells from patients with HIV-1 infection and merit further investigation as an immunomodulatory strategy in an *ex vivo* whole-cell vaccination approach. Additional characterization in animal models to demonstrate the control of viremia and a polyfunctional CTL response in animals challenged with autologous NTP-exposed cells will help identify the mechanisms in play. Successful completion of these pre-clinical studies will lay the foundation for the development of an effective therapeutic vaccination strategy based on NTP.

## Supporting information

S1 Fig GFP expression is robustly stimulated in J-Lat cells (clone 10.6) by PMA treatment. J-Lat cells were treated with 100 nM of phorbol 12-myristate 13-acetate (PMA) (n = 3) for 24 h and then analyzed for GFP expression via flow cytometry. Stimulation with PMA normally induces at least 70% GFP positive cells. Data are presented as mean ± SEM, from three independent experiments. p-values were calculated using unpaired Student’s t-test with Welch’s correction. ***p<0.001.
(DOCX)

S2 Fig HMBG1 release is highly stimulated by NTP exposure. J-Lat cells were exposed to NTP, and supernatants from cultures were collected by centrifugation 24 h later for HMGB1 detection via Western immunoblot analyses. Exposure to 15 s of NTP induced release of HMGB1 in a dose-dependent manner. Mitoxantrone (MTX) at 250 nM was used as a positive control for HMGB1 release.
(DOCX)

S3 Fig GFP-positive J-Lat cell populations predominantly display pro-phagocytic DAMPs. J-Lat cells were exposed to NTP for 15 s and assayed for GFP expression and surface expression of CRT, HSP70, and HSP90 24 h post-exposure. The majority of cells expressing GFP were also double-positive of CRT, HSP7, or HSP90. Data are presented as mean ± SEM from at least three independent experiments with three replicates.
(DOCX)
