# Supplementary material for: Non-thermal plasma modulates cellular markers associated with immunogenicity in a model of latent HIV-1 infection
Source: PLoS One. 2021 Mar 1;16(3):e0247125. doi: 10.1371/journal.pone.0247125 (PMC7920340; doi:10.1371/journal.pone.0247125)
Supplement: S1 Fig — J-Lat cells were treated with 100 nM of phorbol 12-myristate 13-acetate (PMA) (n = 3) for 24 h and then analyzed for GFP expression via flow cytometry. Stimulation with PMA normally induces at least 70% GFP positive cells. Data are presented as mean ± SEM, from three independent experiments. p-values were calculated using unpaired Student’s t-test with Welch’s correction. ***p<0.001. (DOCX) [file pone.0247125.s001.docx]

**S1 Fig. GFP expression is robustly stimulated in J-Lat cells (clone 10.6) by PMA treatment.** J-Lat cells were treated with 100 nM of phorbol 12-myristate 13-acetate (PMA) (n=3) for 24 h and then analyzed for GFP expression via flow cytometry. Stimulation with PMA normally induces at least 70% GFP positive cells. Data are presented as mean ± SEM, from three independent experiments. p-values were calculated using unpaired Student’s t-test with Welch’s correction. ***p<0.001.

**
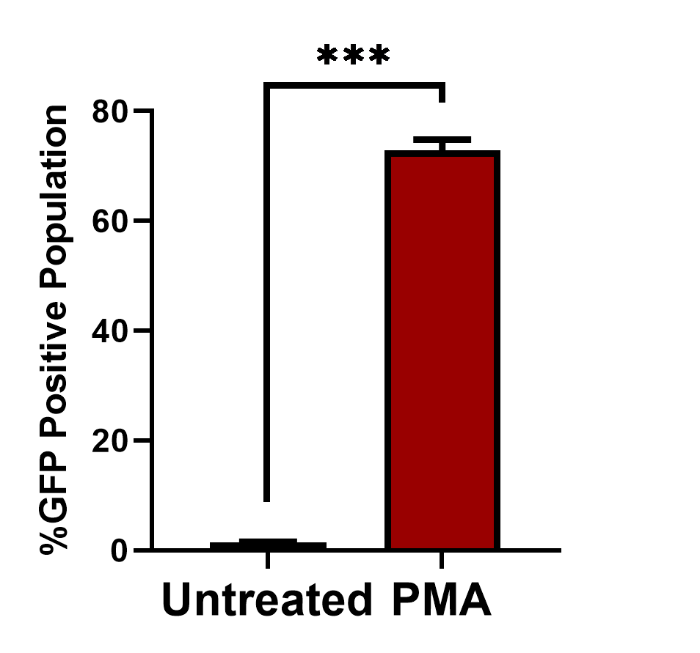
**
