# Supplementary material for: Non-thermal plasma modulates cellular markers associated with immunogenicity in a model of latent HIV-1 infection
Source: PLoS One. 2021 Mar 1;16(3):e0247125. doi: 10.1371/journal.pone.0247125 (PMC7920340; doi:10.1371/journal.pone.0247125)
Supplement: S2 Fig — J-Lat cells were exposed to NTP, and supernatants from cultures were collected by centrifugation 24 h later for HMGB1 detection via Western immunoblot analyses. Exposure to 15 s of NTP induced release of HMGB1 in a dose-dependent manner. Mitoxantrone (MTX) at 250 nM was used as a positive control for HMGB1 release. (DOCX) [file pone.0247125.s002.docx]

**S2 Fig. HMBG1 release is highly stimulated by NTP exposure.** J-Lat cells were exposed to NTP, and supernatants from cultures were collected by centrifugation 24 h later for HMGB1 detection via Western immunoblot analyses. Exposure to 15 s of NTP induced release of HMGB1 in a dose-dependent manner. Mitoxantrone (MTX) at 250 nM was used as a positive control for HMGB1 release.

**
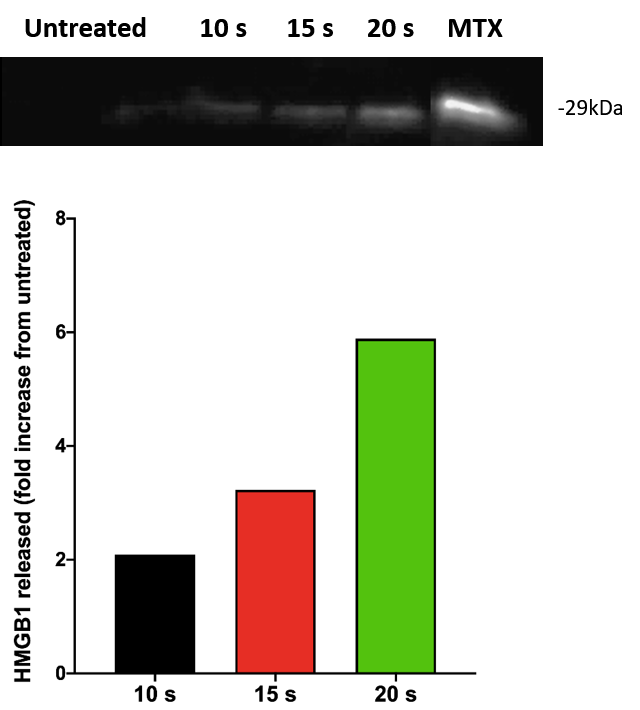
**
