# Supplementary material for: Non-thermal plasma modulates cellular markers associated with immunogenicity in a model of latent HIV-1 infection
Source: PLoS One. 2021 Mar 1;16(3):e0247125. doi: 10.1371/journal.pone.0247125 (PMC7920340; doi:10.1371/journal.pone.0247125)

**S3 Fig. GFP-positive J-Lat cell populations predominantly display pro-phagocytic DAMPs.** J-Lat cells were exposed to NTP for 15 s and assayed for GFP expression and surface expression of CRT, HSP70, and HSP90 24 h post-exposure. The majority of cells expressing GFP were also double-positive of CRT, HSP7, or HSP90. Data are presented as mean ± SEM from at least three independent experiments with three replicates.


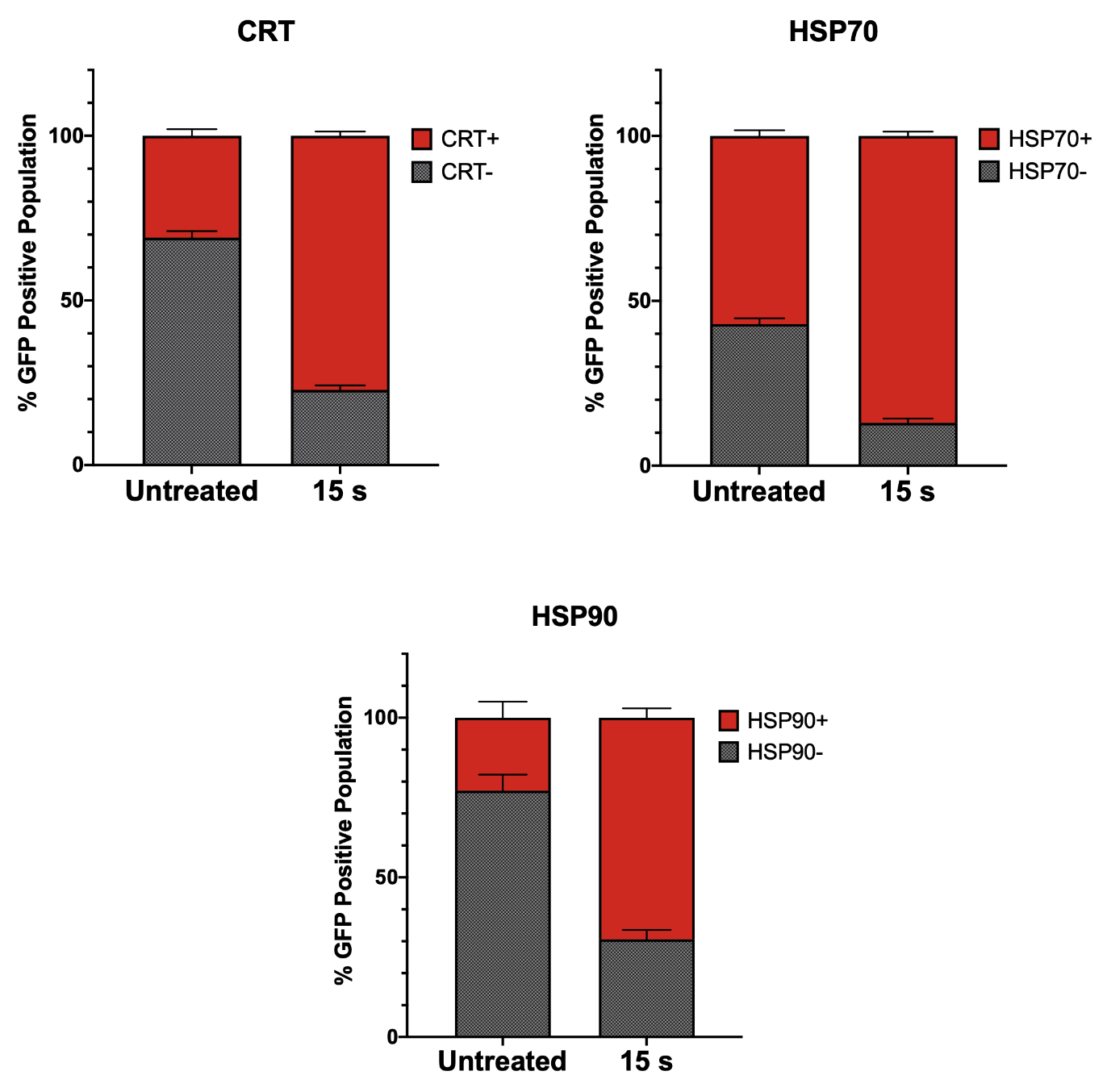

Supplement: S3 Fig — J-Lat cells were exposed to NTP for 15 s and assayed for GFP expression and surface expression of CRT, HSP70, and HSP90 24 h post-exposure. The majority of cells expressing GFP were also double-positive of CRT, HSP7, or HSP90. Data are presented as mean ± SEM from at least three independent experiments with three replicates. (DOCX) [file pone.0247125.s003.docx]
